# Assessment of Cognitive Performance and Psychophysiological Signals in Mental Patients by a Novel Method

**DOI:** 10.1192/j.eurpsy.2024.144

**Published:** 2024-08-27

**Authors:** M. Fűzi, B. Petró, P. Barna, K. Kósa

**Affiliations:** ^1^Department of Behavioural Sciences, University of Debrecen, Faculty of Medicine, Debrecen; ^2^Aviatronic Ltd., Budapest, Hungary

## Abstract

**Introduction:**

Mental disorders often manifest broad cognitive deficits that detrimentally affect daily functioning. Stress indicated by heart rate variability (HRV) has been linked to these cognitive functions.

**Objectives:**

We aimed to develop a new method to assess cognitive performance and simultaneous measurement of psychophysiological signals related to stress and relaxation levels.

**Methods:**

20 adult patients with mental disorders in a rehabilitation program were recruited along with 21 healthy volunteers. A test protocol was carried out with a purpose-developed computerized psychophysiological device. The protocol consisted of a relaxation period; digitized questionnaires on pathological distress (GHQ) and sense of coherence (SOC); gamified cognitive tasks to assess working memory, attention, and decision-making; and a final relaxation period. Acute stress was assessed by heart rate variability measured by a wireless ECG sensor. The inter-beat interval’s root mean square of successive differences (RMSSD) was calculated as an inverse stress measure. Relaxation levels were assessed by the relative power of the alpha frequency band measured by a commercial 4-channel EEG headband. Stress and relaxation levels were compared to the first relaxation period as a baseline.

**Results:**

Patients scored worse than the reference group both regarding distress (d=7, p=0.004) and sense of coherence (d=-8, p=0.047). The cognitive performance of patients was significantly lower (p<0.001) than the reference group for all tasks.

RMSSD at baseline tended to be lower for patients (d=-12.69, p=0.098), reflecting a higher level of physiological stress; 61% of patients started at an elevated stress level compared to 25% of the reference group. In addition, relative alpha levels at baseline were also lower (d=-5.8%, p=0.007) for patients.

Compared to baseline, RMSSD decreased on average to 94% during cognitive assessments in patients and decreased to 91% by the end of the final relaxation. RMSSD decreased to 76% in the reference group and reached a final value of 78% of the baseline. Alpha levels slightly increased among patients during the tasks (103.4%) and then returned close to baseline (99.1%). For the reference group, alpha decreased during the tasks (95.5%) and then slightly increased (97.3%).

**Conclusions:**

Patients displayed heightened distress, reduced sense of coherence, and inferior cognitive scores compared to controls. While starting with higher stress, patients exhibited less elevation in stress during tasks, coupled with alterations in alpha levels, suggesting diminished engagement or focus. Our innovative method could aid in the diagnostics of cognitive performance in mental patients after further measurements for validation.

**Disclosure of Interest:**

None Declared

